# Effects of quantitative trait loci determining testicular weight in DDD/Sgn inbred mice are strongly influenced by circulating testosterone levels

**DOI:** 10.5713/ajas.18.0783

**Published:** 2019-03-07

**Authors:** Jun-ichi Suto, Misaki Kojima

**Affiliations:** 1 Institute of Agrobiological Sciences, National Agriculture and Food Research Organization (NARO), Tsukuba, Ibaraki 305-8634, Japan; 2 Institute of Livestock and Grassland Science, National Agriculture and Food Research Organization (NARO), Tsukuba, Ibaraki 305-0901, Japan

**Keywords:** DDD/Sgn Mice, Quantitative Trait Loci (QTL) Mapping, Testis Weight, Testosterone Level, Whole-exome Sequence Analysis

## Abstract

**Objective:**

Testicular growth and development are strongly influenced by androgen. Although both testis weight and plasma testosterone level are inherited traits, the interrelationship between them is not fully established. Males of DDD/Sgn (DDD) mice are known to have extremely heavy testes and very high plasma testosterone level among inbred mouse strains. We dissected the genetic basis of testis weight and analyzed the potential influence of plasma testosterone level in DDD mice.

**Methods:**

Quantitative trait loci (QTL) mapping of testis weight was performed with or without considering the influence of plasma testosterone level in reciprocal F_2_ intercross populations between DDD and C57BL/6J (B6) mice, thereby assessing the influence of testosterone on the effect of testis weight QTL. Candidate genes for testis weight QTL were investigated by next-generation sequencing analysis.

**Results:**

Four significant QTL were identified on chromosomes 1, 8, 14, and 17. The DDD-derived allele was associated with increased testis weight. The F_2_ mice were then divided into two groups according to the plasma testosterone level (F_2_ mice with relatively “low” and “high” testosterone levels), and QTL scans were again performed. Although QTL on chromosome 1 was shared in both F_2_ mice, QTL on chromosomes 8 and 17 were identified specifically in F_2_ mice with relatively high testosterone levels. By whole-exome sequencing analysis, we identified one DDD-specific missense mutation Pro29Ser in alpha tubulin acetyltransferase 1 (*Atat1*).

**Conclusion:**

Most of the testis weight QTL expressed stronger phenotypic effect when they were placed on circumstance with high testosterone level. High testosterone influenced the QTL by enhancing the effect of DDD-derived allele and diminishing the effects of B6-derived allele. Since Pro29Ser was not identified in other inbred mouse strains, and since Pro29 in *Atat1* has been strongly conserved among mammalian species, *Atat1* is a plausible candidate for testis weight QTL on chromosome 17.

## INTRODUCTION

Testis weight is a physiologically important quantitative trait because of its direct implication in male fertility, i.e., spermatogenic ability. Several lines of evidence suggest that sperm production rate depends on testis weight [1,2]. Besides spermatogenesis, testis weight might be associated with various physiological processes; e.g., mating and aggression behaviors [3]. Interestingly, when lines were selected for testis weight, a correlated response for ovulation rate in females emerged in mice [4] and golden hamsters [5]. This suggests that sex-limited phenotypes are mutually correlated probably by sharing common physiologic pathways [6]. Therefore, elucidating the genetic basis underlying testis weight will be help to reveal various physiologic mechanisms of reproductive processes in males as well as females.

Since testis weight varies widely among inbred mouse strains (pared testis weight ranged about 100 to 300 mg), several studies pursuing testis weight genes have been performed [3,7–10]. Since males of the inbred mouse DDD/Sgn strain are known to have extremely heavy testes among inbred mouse strains, we have previously performed quantitative trait loci (QTL) mapping analyses in two F_2_ intercross populations using DDD mice; i.e., F_2_ mice between DDD and DH/Sgn (DH) inbred mice [9] and F_2_ mice between DDD and CBA/N (CBA) inbred mice [10]. In DDD×DH F_2_ mice, we identified significant testis weight QTL on chromosome (Chr) 9 (*Twdq1*) and suggestive QTL on Chrs 4, 5, 14, 17, and 18. In DDD×CBA F_2_ mice, we identified significant QTL on Chrs 1 (*Twdq2*) and X (*Twdq3*) and suggestive QTL on Chrs 2, 8, 9, 11, 13, and 14. The localization of QTL differed substantially between two crosses, and if any, there were some overlapping QTL. This finding suggests that there would be additional, yet-to-be-identified genes, which contribute to the heavy testes of DDD mice. In the present study, as a third QTL mapping study aimed at genetically dissecting the heavy testes in DDD mice, we produced and analyzed F_2_ intercross populations between DDD and C57BL/6J (B6) inbred mice.

In addition to heavy testes, DDD males are known to have very high blood testosterone level [11,12]. Indeed, while the average plasma testosterone level in B6 males was 0.49±0.18 ng/mL, that in DDD males was 13.07±3.12 ng/mL [12]. Testicular growth and development is strongly influenced by androgen [13,14]. Although both testis weight and plasma testosterone level are inherited traits, interrelationship between them is not fully established. We have previously performed QTL mapping for plasma testosterone level in F_2_ male mice between DDD and B6 [12] but did not detect any significant QTL. Even though the genetic basis of plasma testosterone level in DDD mice is unclear, we hypothesize that high blood testosterone level influence extremely heavy testes in DDD mice. Therefore, in this study, we first performed QTL mapping for testis weight and then investigated the influence of plasma testosterone level on the effect of the testis weight QTL.

We further performed whole-exome sequencing analysis in DDD mice to identify genes underlying testis weight QTL. Since extremely heavy testes are a prominent phenotype of DDD mice among inbred mouse strains, it was expected that there would be candidate genes carrying DDD-specific variations.

## MATERIALS AND METHODS

### Animal ethics statement

All animal procedures were reviewed and approved by the Institutional Animal Care and Use Committee of the NIAS (authorization number H25-001).

### Mice

The inbred mouse strains DDD and B6 were maintained at the National Institute of Agrobiological Sciences (NIAS, Tsukuba, Japan). Reciprocal crosses between DDD and B6 strains produced DB (♀DDD×♂B6) F_1_ and BD (♀B6×♂DDD) F_1_ mice, both of which were intercrossed to produce DB F_2_ (n = 150) and BD F_2_ (n = 150) male mice. Testicular weight was determined in DDD, B6, and F_1_ mice at the age of 13 to 14 weeks (90±5 days after birth), and in F_2_ mice at the age of 11 to 12 weeks (71 to 80 days after birth).

All mice were weaned at 4 weeks of age and four to five mice were housed together in each cage during the experiments. All mice were maintained in a specific pathogen-free facility with a regular light cycle, controlled temperature, and humidity. Food (CRF-1; Oriental Yeast Co., Ltd., Tokyo, Japan) and water were freely available throughout the experimental period.

### Testosterone and testis weight analyses

The plasma testosterone level was determined in parental, F_1_ and F_2_ mice, as previously described [12]. The paired testes were weighed on an electric balance to the nearest milligram. The weight of the paired testes is simply designated as “testis weight.”

### Genotyping

Microsatellite sequence length polymorphisms were identified by electrophoresis after polymerase chain reaction (PCR) amplification of genomic DNA. The PCR amplification was carried out using the Thermal Cycler Dice (TaKaRa Bio Inc., Shiga, Japan). The PCR products were separated on 10% polyacrylamide gel (Nacalai Tesque Inc., Kyoto, Japan) and were visualized by ethidium bromide (Nacalai Tesque, Japan) staining. In total, 117 microsatellite loci were genotyped. Their chromosomal positions were retrieved from Mouse Genome Informatics (MGI, http://www.jax.org). Because the chromosomal positions of six markers were unavailable, they were determined based on our own linkage map.

### Quantitative trait loci analysis

Normality of testis weight was assessed using the Shapiro–Wilk W test (JMP13, SAS Institute Japan Inc., Tokyo, Japan). QTL mapping was performed using R/QTL version 1.38–4 [15,16]. Single-QTL scans were performed by computing at 1 cM intervals across the entire genome with or without using the lineage (i.e., the direction of the cross) effect as a covariate. First, we included the lineage effect as an additive covariate in the single-QTL scan, if the lineage effect had a strong effect on testis weight. Next, we included the lineage effect as an interactive covariate because the effect of QTL may vary with the covariate (i.e., QTL×covariate interaction). Threshold logarithm of the odds (LOD) scores for significant (p<0.05) and suggestive (p<0.63) linkages were determined by performing 1,000 permutations [17,18]. After single-QTL scans, two-QTL scans were performed to identify pairwise interactions. In this case, we strictly adhered to a recommended threshold [15]. Finally, the covariates and the combined effects of all QTL, including those that were significant and suggestive, were assessed using multiple-QTL models [19].

### Whole-exome sequence analysis

To identify nonsynonymous single-nucleotide variation (nsSNV) and/or insertion-deletion in the coding regions of candidate genes, whole-exome sequence analyses were performed. Genomic DNA was extracted from the tail of DDD mice using a genomic DNA purification kit (Wizard Genomic DNA Purification Kit, Promega KK, Tokyo, Japan) and was submitted to Filgen, Inc. (Nagoya, Aichi, Japan) for exome capture and sequencing. Sequence reads were mapped to the mouse reference genome (GRCm38, mm10). Read mapping and variant analyses were performed using CLC Genomics Workbench 7.0.4 and 8.5.1 (Filgen, Japan).

### Alpha tubulin acetyltransferase 1 sequencing

Genomic DNA from inbred strains including DDD, B6, DH/Sgn, and CBA/N were extracted according the protocol described above. PCR was performed using *Atat1*-specific primers (F: 5′-ttcccgttcgatgtggat and R: 5′-gtaaataacgtgccggttgc). The PCR product was purified by PCR purification kit (LaboPass PCR, Hokkaido System Science., Ltd. Sapporo, Japan) and submitted to Hokkaido System Science for direct sequence with these primers.

### Statistical analysis

Statistical analyses were performed using JMP 13 (SAS Institute, Japan). Testis weight was represented as the mean± standard error of the mean (mg). Statistical differences between two groups were analyzed using Student’s or Welch’s *t*-test. For statistical comparison among more than two groups, the Tukey–Kramer honest significant difference test was used. Statistical significance is defined when p values are less than 0.05.

## RESULTS

### Localization of testis weight quantitative trait loci

Testis weight in DDD males (n = 28) was significantly higher than that in B6 males (n = 16) (277.6±3.7 mg vs 206.2±5.3 mg, p<0.0001). A clear lineage effect was identified in F_1_ mice; i.e., testis weight in BD F_1_ males (n = 12) was significantly higher than that in DB F_1_ males (n = 15) (301.5±4.8 mg vs 279.7±3.7 mg, p<0.002).

**Figure 1:**
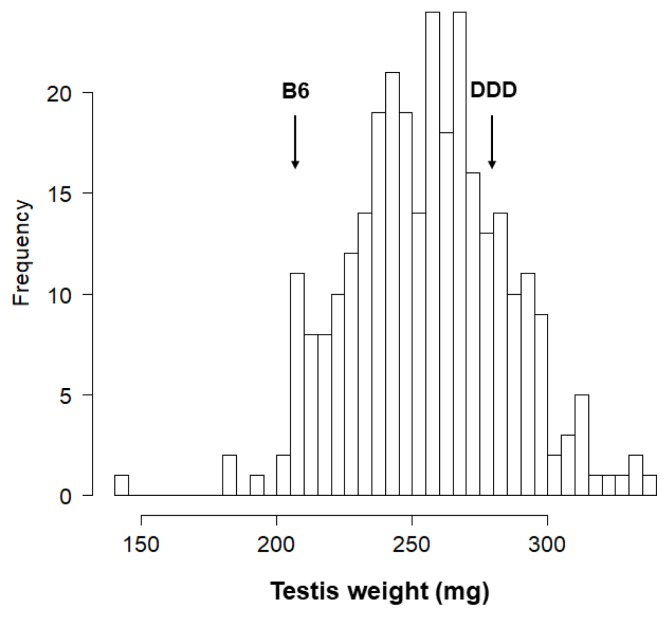
A histogram showing the distribution of testis weight in 297 F_2_ mice (data from BD [♀B6×♂DDD] F_2_ and DB [♀DDD×♂B6] F_2_ mice are combined). The mean±SEM is 255.8±1.7 mg (The mean±SEM testis weight in BD F_2_ mice is 257.0±2.3 mg and that in BD F_2_ mice is 254.6±2.4 mg. The difference was not statistically significant). The average testis weight of parental B6 and DDD strains are indicated by arrows. SEM, standard error of the mean.

Figure 1 depicts a histogram showing the distribution of testis weight in 297 F_2_ male mice (We failed to determine the testis weight in one BD F_2_ and one DB F_2_ mouse. In addition, we excluded one DB F_2_ mouse from the analysis because the paired testis weight of this mouse was abnormally low; i.e., only 76.8 mg. Thus, we phenotyped 297 F_2_ mice but genotyped 300 F_2_ mice). The testis weight followed a normal distribution. The average testis weight was 255.8±1.7 mg. Although the difference between BD F_2_ and DB F_2_ males was not statistically significant (257.0±2.3 mg vs 254.6±2.4 mg, p>0.4), we included the lineage effect as an additive covariate in the subsequent single-QTL scan.

**Figure 2:**
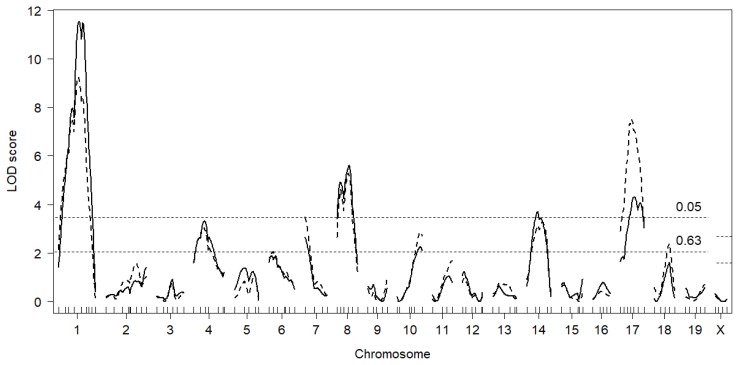
Genome-wide LOD score plot for testis weight when the lineage effect was included as an additive covariate (solid lines) and when the body weight was included as an additive covariate (dashed lines). The x-axis represents chromosomal and microsatellite marker positions and the y-axis represents LOD scores. Horizontal broken lines indicate the genome-wide threshold LOD score for significant (p<0.05) and suggestive (p<0.63) linkage. Threshold LOD scores for significant and suggestive quantitative trait loci were 3.56 and 2.10 for autosomes and 2.73 and 1.48 for Chr X, respectively. LOD, logarithm of the odds.

A LOD score plot for testis weight in F2 males is shown in Figure 2 (solid lines). As shown in Table 1, four significant QTL were identified on Chr 1@57.5 cM (*Twdq2*: this locus was coincidental with that identified in DDD×CBA F_2_ mice [10]; accordingly, the same QTL symbol was assigned), Chr 8@41.5 cM (*Twdq4*), Chr 14@32.3 cM (*Twdq5*), and Chr 17@36.1 cM (*Twdq6*), along with three suggestive QTL on Chr 4@33.1 cM (*Twdq7*: QTL symbol was assigned to this suggestive QTL because a significant testis weight QTL has been mapped to this region [3]), Chr 7@2.0 cM, and Chr 10@61.5 cM (*Twdq8*: QTL symbol was assigned to this suggestive QTL because a significant testis weight QTL has been mapped to this region [3]). The DDD allele was associated with increased testis weight at all QTL except for the *Twdq8* on Chr 10. We next performed QTL mapping by including the lineage effect as an interactive covariate, but found no significant QTL×covariate interactions (in other words, there were no QTL whose effect differed significantly between BD F_2_ and DB F_2_ males). Two-QTL scans with R/QTL revealed a possible pairwise interaction between Chr 1 and Chr 8; however, the LOD score for interaction (lod.int) was substantially lower (2.58) than the recommended threshold value (i.e., 6.3). Multiple regression analysis indicated that the detected QTL explained 41.4% of the testis weight variation (Table 2).

In the above analysis, we found strong evidence for *Twdq2* on Chr 1. Accordingly, we next performed QTL mapping by including the nearest marker (*D1Mit102*) for *Twdq2* as an additive covariate. While the LOD score on Chr 1 shrank to 1.1, the LOD scores for the remaining significant QTL, particularly those for *Twdq4* (Chr 8) and *Twdq5* (Chr 14), were increased to 6.2 and 4.8, respectively. In contrast, the LOD score for *Twdq6* (Chr 17) was changed subtly (LOD score 4.4) (data not shown). One additional suggestive QTL was identified on Chr 5 (Table 1).

We also performed a single-QTL scan by including body weight as an additive covariate. Since the suggestive QTL on Chr 7 was identified as significant (Table 1), we newly assigned the QTL symbol *Twdq9* to this QTL. Clearly, the LOD score for *Twdq6* (Chr 17) was substantially increased to 7.7 (Figure 2, dashed lines). One additional suggestive QTL was identified on Chr 18 (Table 1).

### Influence of testosterone level on the localization of quantitative trait loci

**Figure 3:**
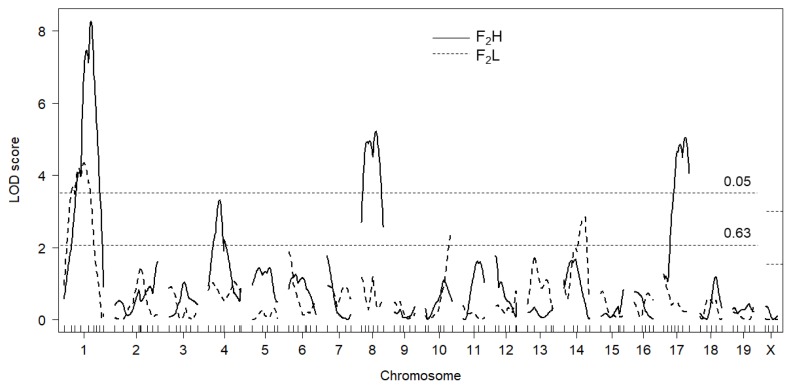
Genome-wide LOD score plot of the QTL for testis weight when the F_2_H (solid lines) and F_2_L (dashed lines) mice were analyzed separately. The x-axis represents the chromosomal and microsatellite marker position, and the y-axis represents the LOD score. Horizontal broken lines indicate the genome-wide threshold LOD score for significant (p<0.05) and suggestive (p<0.63) linkage. Threshold LOD scores for significant and suggestive QTL were 3.37 and 2.08 for autosomes and 2.72 and 1.50 for Chr X, respectively. LOD, logarithm of the odds; QTL, quantitative trait loci.

**Figure 4:**
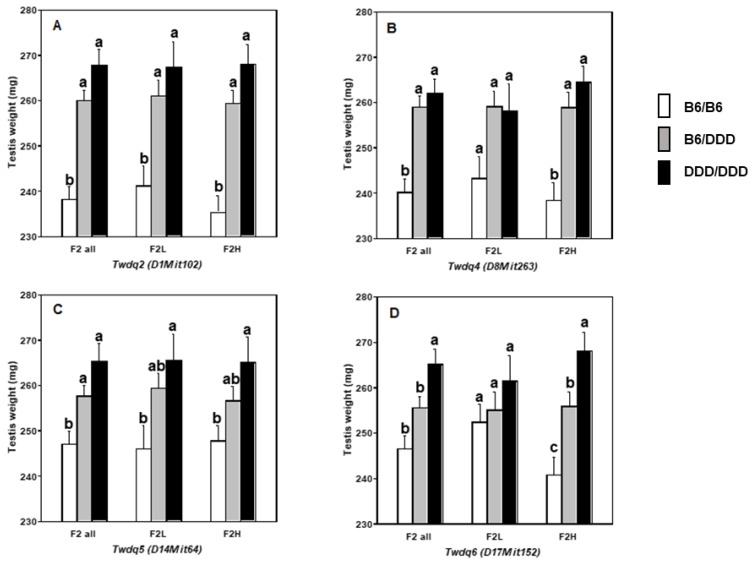
Comparison of allele effect of significant QTL among all F_2_, F_2_L, and F_2_H mice. The x-axis represents the genotype at the microsatellite marker nearest QTL, and the y-axis represents average testis weight. Mice with different superscripts denote the presence of significant difference. Statistical difference between mice with three genotypes within each F_2_ group (i.e., F_2_ all, F_2_L, and F_2_H) was identified by Tukey–Kramer honestly significant difference tests. Error bars indicate standard error of means. QTL, quantitative trait loci.

Next, we analyzed testis weight by taking circulating testosterone level into consideration, because DDD male has extremely high plasma testosterone level. The F_2_ mice were then divided into two groups according to the plasma testosterone level; i.e., F_2_ mice with less than 0.49 ng/mL testosterone (an average testosterone level in B6 male) were designated as F_2_L (F_2_ with “low” testosterone, n = 124, an average and a median testosterone level were 0.33±0.01 ng/mL and 0.34 ng/mL, respectively) mice and F_2_ mice with more than 0.49 ng/mL testosterone were as F_2_H (F_2_ with “high” testosterone, n = 176, an average and a median testosterone level were 7.84±0.78 ng/mL and 2.02 ng/mL, respectively) mice. The difference in testosterone level between F_2_L and F_2_H mice was statistically significant (p<0.0001) when judged by either parametric (Student’s *t*-test) or non-parametric (Wilcoxon rank sum test) analysis. On the other hand, there was no significant difference in testis weight in F_2_L and F_2_H mice (256.1±2.6 mg vs 255.6±2.2 mg, p>0.8). Single-QTL scans were performed for F_2_L and F_2_H mice separately. In F_2_L mice, one significant QTL was identified Chr 1@52.5 cM, along with two suggestive QTL on Chr 10@64.5 cM and Chr 14@53.3 cM (Table 3; Figure 3). On the other hand, in F_2_H mice, three significant QTL were identified on Chr 1@68.5 cM, Chr 8@45.5 cM, and Chr 17@52.1 cM, along with one suggestive QTL on Chr 4@33.1 cM. Substantial difference in LOD scores between F_2_L and F_2_H mice was found for QTL on Chrs 1, 4, 8, and 17. Notably, the LOD score for QTL on Chr 17 in F_2_H mice was higher than that identified in all F_2_ mice (Table 1). Although QTL on Chr 1 explained similar variance in F_2_L and F_2_H mice, QTL identified in F_2_H mice accounted for slightly more variance compared with those identified in F_2_L mice (Table 2). Allele effect of significant QTL was investigated in all F_2_, F_2_L, and F_2_H mice (Figure 4). Except for *Twdq5* on Chr 14 (Figure 4C), high testosterone level tended to be associated with increasing the effect of the DDD-derived allele and with decreasing the effect of the B6-derived allele. Testosterone level appeared not to influence the effect of heterozygotes.

### Candidate gene identification for *Twdq6* on Chr 17

**Figure 5:**
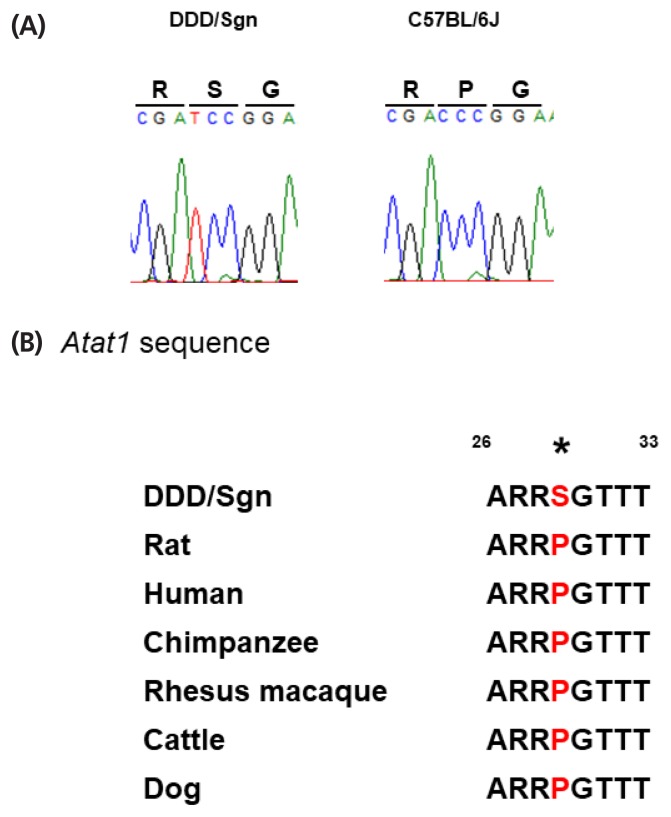
*Atat1* sequence analysis. (A) Sequencing chromatograms for *Atat1* from DDD/Sgn, C57BL/6J, DH/Sgn, and CBA/N mice showing C>T polymorphism. (B) *Atat1* sequence alignment from various species with the novel polymorphism in red. *Atat1*, alpha tubulin acetyltransferase 1.

By submitting the term “abnormal testis weight” as a query to the MGI database (Mammalian Phenotype Browser), we retrieved 25 genes that were localized within 95% confidence intervals (CIs) of four significant single-QTL on Chrs 1, 8, 14, and 17 (Table 4). In most cases, testis weight was reduced in the mutant mice of these genes; only 2 of 25 mutants (protein tyrosine phosphatase, receptor type, V [*Ptprv*] and TBC domain family, member 4 [*Tbc1d4*]) were associated with increased testis weight. Since DDD mice have extremely heavy testis relative to other inbred mice, we assumed that there are DDD-specific genetic variations. To identify such genetic variations, we performed whole-exome sequence analysis. We identified nsSNVs in eight genes, i.e., tudor domain containing 5 (*Tdrd5*), neuregulin 1 (*Nrg1*), nanos C2HC-type zinc finger 3 (*Nanos3*), synaptonemal complex central element protein 2 (*Syce2*), *Tbc1d4*, mutS homolog 5 (*Msh5*), *Atat1*, and follicle stimulating hormone receptor (*Fshr*), but none of them except for one that detected for *Atat1* were DDD-specific. p.Pro29Ser resulting from a c.85C>T substitution (Chr 17:35909951) in *Atat1* seems to be novel because no single nucleotide polymorphism (SNP) ID has been assigned, and because no SNP were known at this chromosomal location in other inbred mouse strains including 129S1/SvImJ, A/J, AKR/J, BTBR T^+^ Itp3^tf^/J, C3H/HeJ, C57BL/6J, CAST/EiJ, CBA/J, DBA/1J, DBA/2J, FVB/NJ, I/LnJ, KK/HIJ, MOLF/EiJ, NOD/ShiLtJ, NZB/BINJ, NZO/HILtJ, PWK/PhJ, RF/J, SPRET/EiJ, and WSB/EiJ according to “Mouse SNP retrieval utility” by Mouse Phenome Database (Figure 5A). Furthermore, Pro29 was well conserved among mammalian species including human, rat, chimpanzee, rhesus macaque, cattle, and dog (Figure 5B).

## DISCUSSION

This study identified nine QTL, of which five were significant and four were suggestive. The result was satisfactory because the number of QTL underlying testis weight was thought to be small [7,20]. Seven of the nine QTL, i.e., *Twdq2* (Chr 1), *Twdq7* (Chr 4), *Twdq4* (Chr 8), *Twdq5* (Chr 14), *Twdq6* (Chr 17), and the suggestive QTL on Chrs 5 and 18, had also been identified in either of our preceding studies using DDD mice [9, 10]. Therefore, only *Twdq9* (Chr 7) and *Twdq8* (Chr 10) were novel in a series of QTL mapping studies using DDD mice. Based on the results of three studies, most of the QTL underlying high testis weight in DDD mice might have been identified. Notably, the DDD-derived allele was associated with higher testis weight at most QTL.

The lineage effect was observed in the F _1_ populations, i.e., BD F_1_ mice had significantly higher testis weight than DB F_1_ mice. The lineage effect was also found in our previous studies; e.g., DH×DDD F_1_ mice had significantly higher testis weight than that of the DDD×DH F_1_ mice [9], and CBA×DDD F_1_ mice had significantly higher testis weight than that of the DDD×CBA F_1_ mice [10]. We attribute the lineage effect to the difference in the genes on the Chr Y because the F_1_ mice carrying Y^DDD^ had significantly higher testis weight than the reciprocal F_1_ mice in all crosses. There is experimental evidence supporting the contribution of Chr Y to testis weight in mice [21]. Our studies in Y-consomic strains clearly showed the effect of Chr Y; Chr Y^DDD^ produced significantly heavier testes than did Chr Y^B6^ [9,10]. Further evidence was that DDD mice had significantly heavier testis than DDD-Chr Y^CBA^ mice (296.0±4.4 mg vs 252.2±3.8 mg, p<0.0001), although the body weight did not significantly differ between the two strains (32.3±0.4 g vs 31.8±0.3 g, p>0.3) (unpublished data). It was suggested that the native Y^DDD^ was indispensable to sustain high testis weight in DDD mice. The effect of Chr Y appeared to be independent of the effect of autosomes.

To control the effect of body weight, we analyzed testis weight by including body weight as an additive covariate. Accordingly, we re-analyzed previously published testis weight data in the DDD×DH F_2_ mice [9] and DDD×CBA F_2_ mice [10] by including body weight as an additive covariate. As a result, two of four suggestive QTL identified in DDD×DH F_2_ mice, i.e., QTL on Chrs 14 and 17, were identified as significant QTL (the maximum LOD scores for these QTL were 4.8 and 4.7, respectively). The result suggests that these QTL may have an indirect effect on testis weight, acting through the body weight [15]. The QTL on Chr17 identified in DDD× DH F_2_ mice might be allelic to that identified in this study because of considerable increase of LOD score after the inclusion of body weight as an additive covariate. In contrast, it was uncertain whether the QTL on Chr 14 identified in the two crosses were allelic, because the LOD score for *Twdq5* identified in the present study was not substantially changed after the inclusion of body weight as an additive covariate.

Results of separate F _2_ analyses (i.e., F_2_L and F_2_H mice) strongly suggested that we should take the endocrinological background of the mice into consideration when assessing the effect of genes on testis weight. As it was particularly evident for the QTL on Chr 17, “high” testosterone level altered the mode of inheritance of QTL allele. In other words, “high” testosterone level tended to be associated with increasing the effect of the DDD-derived allele and with decreasing the effect of the B6-derived allele. We considered the possible mechanism for this phenomenon to be that, testosterone level might change the magnitude of expression level of genes underlying QTL. Indeed, testosterone is known to regulate gene expression levels [22,23]. Thus, high testosterone might be associated with up-regulation of the DDD-derived genes and was associated with down-regulation of the B6-derived genes. Otherwise, high testosterone might sensitize the cells or cellular receptors, on which the genes underlying QTL act. In this context, we were interested in the genetic basis of plasma testosterone level. Like other blood components, testosterone level is suggested to be genetically determined. Serum testosterone level is inherited in an autosomal dominant mode in pig breeds Meishan and Landrace [22], and significant QTL was identified in White Duroc×Chinese Erhualian resource population [24]. However, the mode of inheritance of circulating testosterone level was ambiguous in mice, and we could not identify any significant QTL [12]. In part, this result is associated with the extensive variation in the plasma testosterone levels in DDD mice. Since the episodic testosterone secretion is known in mice [25,26], variable testosterone level even in an inbred mouse strain might not be surprising. We should be cautious about species difference when interpreting the experimental results regarding the blood testosterone levels.

By searching MGI database, we found 25 candidate genes that potentially influence testis weight within 95% CIs for four significant single-QTL on Chrs 1, 8, 14, and 17 (Table 4). Interestingly, testis weight was decreased in most mutants of the candidate genes. Dysfunctional gene mutations tend to be associated with lower testis weight; thus, the higher testis weight of DDD mice might be a consequence of altered gene functions. We performed whole-exome sequencing in DDD mice on the assumption that the higher testis weight of DDD mice is caused by coding-region variants, which are specific to the DDD strain. The analysis identified nsSNV that differed between DDD and B6 mice in 8 of 25 candidate genes: *Tdrd5*, *Nrg1*, *Nanos3*, *Syce2*, *Tbc1d4*, *Msh5*, *Atat1*, and *Fshr* (Table 4). However, most of nsSNV identified for these genes were not DDD strain-specific. For example, although there were six nsSNVs in *Tdrd5*, all were also found in many other inbred mouse strains. In particular, two of six nsSNV in *Tdrd5* resulting in Thr396Ala and Ile111Met were also identified in NZB/BINJ strain, which have extremely heavy testes like DDD [3]. However, no significant testis weight QTL was identified on Chr 1 in C57BL/6ByJ × NZB/BINJ F_2_ mice, suggesting that these were unlikely to be causative of *Twdq2*. Similarly, two nsSNVs in *Tbc1d4* resulting in Ile834Val and Arg659Gly were identified in NZB/BINJ, but no significant testis weight QTL was identified on Chr 14 in abovementioned F_2_ mice [3]; therefore, these were unlikely to be causative of *Twdq5*. Although the nsSNVs in *Fshr* are plausible candidates underlying *Twdq6*, these nsSNVs were also found in other inbred mouse strains including a strain with very light testes such as CAST/EiJ.

One nsSNV, Pro29Ser, identified in *Atat1* appears to be novel, given that no SNP ID has yet been assigned. Accordingly, we searched for this nsSNV among the various inbred mouse strains with consequent that this nsSNV was not shared by any other strains. We then investigated *Atat1* sequence in other mammalian species and found that Pro29 was well conserved. Thus, we concluded that Ser29 was a mutation occurred specifically in DDD mice. A targeted disruption mutation in *Atat1* resulted in reduction of testis weight in mice [27]. Therefore, Pro29Ser was unlikely to be associated with loss or hypofunction of this gene, if this gene is a causative of *Twdq6*. Although further-in-depth *in vivo* studies are necessary for validating the function of this mutation, this was an important finding to understand the molecular basis of mechanisms underlying high testis weight in mice. Taken together, results of the present study provide insights into genetic and endocrinological mechanisms determining testis weight in mice.

## Figures and Tables

**Table 1:** Significant and suggestive QTL identified by the genome-wide scan of F2 mice

QTL1)	Chr	Peak cM	95% CI2)	LOD3)	Nearest marker	High strain4)
*Twdq2*	1	57.5	49.5–72.5	**11.5**	*D1Mit102*	DDD
*Twdq7*	4	33.1	8.1–61.1	3.3	*D4Mit178*	DDD
	7	2.0	2.0–20.2	2.7	*D7Mit178*	DDD
*Twdq4*	8	41.5	14.5–50.1	**5.6**	*D8Mit263*	DDD
*Twdq8*	10	61.5	35.5–66.7	2.2	*D10Mit180*	B6
*Twdq5*	14	32.3	16.3–57.3	**3.7**	*D14Mit64*	DDD
*Twdq6*	17	36.1	17.1–60.7	**4.3**	*D17Mit152*	DDD
By including *D1Mit102* as an additive covariate						
	5	41.6	17.8–72.8	2.5	*D5Mit113*	DDD
By including body weight as an additive covariate						
*Twdq9*	7	2.0	2.0–16.0	**3.5**	*D7Mit178*	DDD
	18	45.9	28.9–55.9	2.4	*D18Mit188*	DDD

QTL, quantitative trait loci; CI, confidence interval; LOD, logarithm of the odds.

Lineage effect was included as an additive covariate.

1) QTL symbols were assigned if they were significant, or if they were suggestive but identified as significant at least once previously in different genetic crosses.

2) 95% CI was defined by a 1.5–LOD drop.

3) LOD scores for significant QTL are indicated in bold.

4) High strain-derived alleles were associated with higher testis weight.

**Table 2:** Multiple regression analysis for testis weight

Mice	Chr (cM)	df1)	Variance (%)2)	F value
All F_2_3)	Chr1@57.5	2	13.7	33.0
	Chr4@33.1	2	2.0	4.8
	Chr7@2.0	2	3.1	7.4
	Chr8@41.5	2	4.9	11.8
	Chr10@61.5	2	1.8	4.4
	Chr14@32.3	2	5.6	13.5
	Chr17@37.1	2	4.5	10.7
	Total	14	41.4	-
F_2_L	Chr1@52.5	2	12.7	10.5
	Chr10@64.5	2	5.6	4.7
	Chr14@53.3	2	8.3	6.9
	Total	6	29.9	-
F_2_H	Chr1@68.5	2	12.0	16.4
	Chr4@33.1	2	2.7	3.7
	Chr8@45.5	2	5.8	8.0
	Chr17@52.1	2	9.0	12.4
	Total	8	39.8	-

1) Degrees of freedom.

2) Percentage of total F_2_ phenotypic variance.

3) Lineage effect was included as a covariate.

**Table 3:** Significant and suggestive QTL identified by the genome-wide scan of separate F_2_ mice

Mice	QTL1)	Chr	Peak cM	95% CI2)	LOD3)
F_2_L	*Twdq2*	1	52.5	16.5–72.5	**4.3**
	*Twdq8*	10	64.5	45.5–66.7	2.4
	*Twdq5*	14	53.3	22.3–62.3	2.8
F_2_H	*Twdq2*	1	68.5	51.5–75.5	**8.3**
	*Twdq7*	4	33.1	15.1–52.1	3.3
	*Twdq4*	8	45.5	15.5–58.5	**5.2**
	*Twdq6*	17	52.1	25.1–60.7	**5.1**

QTL, quantitative trait loci; CI, confidence interval; LOD, logarithm of the odds.

1) QTL symbols were assigned if they were significant, or if they were suggestive but identified as significant at least once previously in different genetic crosses.

2) 95% CI was defined by a 1.5–LOD drop.

3) LOD scores for significant QTL are indicated in bold.

**Table 4:** Candidate genes, mutant phenotypes, and nsSNV identified for candidate genes of significant QTL

Chr	Position cM	Gene	Testis weight in mutants	Location	Nucleotide change	Amino acid change	db SNP ID
1	57.91	*Kiss1*	Decrease	-	-	-	-
	57.94	*Snrpe*	Decrease	-	-	-	-
	58.24	*Ptprv*	Increase	-	-	-	-
	61.45	*Aspm*	Decrease	-	-	-	-
	67.71	*Tdrd5*	Decrease	Chr 1: 156262881	c.2594C>G	p.Pro865Arg	rs49650703
				Chr 1: 156263413	c.2495C>T	p.Ala832Val	rs13476193
				Chr 1: 156267355	c.2177G>A	p.Gly726Glu	rs48483855
				Chr 1: 156270646	c.2080G>A	p.Asp694Asn	rs30769841
				Chr 1: 156285523	c.1186A>G	p.Thr396Ala	rs31654838
				Chr 1: 156301806	c.333A>G	p.Ile111Met	rs32356404
	71.56	*Slc19a2*	Decrease	-	-	-	-
8	18.75	*Nrg1*	Decrease	Chr 8: 31818081	c.2050T>A	p.Leu684Ile	rs32559738
	20.59	*Tex15*	Decrease	-	-	-	-
	23.89	*Cnot7*	Decrease	-	-	-	-
	26.87	*Ing2*	Decrease	-	-	-	-
	40.45	*Nanos3*	Decrease	Chr 8: 84176519	c.13A>G	p.Asn5Asp	rs38027221
	41.25	*Syce2*	Decrease	Chr 8: 848722541	c.13G>A	p.Gly5Arg	rs32744209
14	18.79	*Tkt*	Decrease	-	-	-	-
	25.36	*Otx2*	Decrease	-	-	-	-
	27.98	*Bcl2l2*	Decrease	-	-	-	-
	36.32	*Piwil2*	Decrease	-	-	-	-
	37.62	*Fndc3a*	Decrease	-	-	-	-
	50.09	*Tbc1d4*	Increase	Chr 14: 101458822	c.2500A>G	p.Ile834Val	rs48744612
				Chr 14: 101477059	c.1975A>G	p.Arg659Gly	rs235588405
17	18.57	*Msh5*	Decrease	Chr 17: 35028832	c.2411C>A	p.Thr804Lys	rs33561826
				Chr 17: 35031264	c.1693A>G	p.Met565Val	rs50143709
	18.59	*Tnf*	Decrease	-	-	-	-
	18.75	*Atat1*	Decrease	Chr 17: 35909951	c.85C>T	p.Pro29Ser	–
	25.86	*Dazl*	Decrease	-	-	-	-
	29.4	*Safb*	Decrease	-	-	-	-
	58.35	*Lhcgr*	Decrease	-	-	-	-
	58.76	*Fshr*	Decrease	Chr 17: 88985240	c.2009C>A	p.Pro670His	rs107828637
				Chr 17: 88985243	c.2006A>G	p.Asn669Ser	rs108743480
				Chr 17: 88986290	c.959G>A	p.Ser320Asn	rs51040992

nsSNV, nonsynonymous single-nucleotide variation; QTL, quantitative trait loci; SNP, single nucleotide polymorphism; *Kiss1*, KiSS-1 metastasis-suppressor; *Snrpe*, small nuclear ribonucleoprotein E; *Ptprv*, protein tyrosine phosphatase, receptor type, V; *Aspm*, abnormal spindle microtubule assembly; *Tdrd5*, tudor domain containing 5; *Slc19a2*, solute carrier family 19 (thiamine transporter), member 2; *Nrg1*, neuregulin 1; *Tex15*, testis expressed gene 15; *Cnot7*, CCR4-NOT transcription complex, subunit 7; *Ing2*, inhibitor of growth family, member 2; *Nanos3*, nanos C2HC-type zinc finger 3; *Syce2*, synaptonemal complex central element protein 2; *Tkt*, transketolase; *Otx2*, orthodenticle homeobox 2; *Bcl2l2*, BCL-like 2; *Piwil2*, piwi-like RNA-mediated gene silencing 2; *Fndc3a*, fibronectin type III domain containing 3A; *Tbc1d4*, TBC1 domain family, member 4; *Msh5*, mutS homolog 5; *Tnf*, tumor necrosis factor; *Atat1*, alpha tubulin acetyltransferase 1; *Dazl*, deleted in azoospermia-like; *Safb*, scaffold attachment factor B; *Lhcgr*, luteinizing hormone/choriogonadotropin receptor; *Fshr*, follicle stimulating hormone receptor.
